# AGING IN COMMUNITY MODELS IN THE US: FRAMEWORKS FOR COMMUNITY SUPPORTIVE NETWORK DIALOGS BETWEEN WEST AND EAST

**DOI:** 10.1093/geroni/igac059.1364

**Published:** 2022-12-20

**Authors:** Su-I Hou

**Affiliations:** University of Central Florida, Orlando, Florida, United States

## Abstract

Aging in Community (AIC) is the preferred way to age. This session shares promising AIC models in the U.S. and analyzed model characteristics. Four models were identified with the potential to achieve person-environment (P-E) fit, including village, naturally occurring retirement community (NORC), cohousing, and university-based retirement community (UBRC). Empirical studies show these AIC models enhance social support and improving older adult's well-being, remain independence at homes, and community social and civic participation. Each model has a unique way to help community-dwelling older adults with their aging needs. This session aims to stimulate dialogs on opportunities and challenges of forming AIC supportive networks considering Eastern versus Western cultural and societal differences. Promising U.S. AIC models shared will serve as conceptual frameworks to facilitate discussions on various model’s strengths and weakness and how they might be adapted in Taiwan’s society considering eastern cultural values, societal norms, and healthcare system. Examples of Taiwan versions of these similar promising U.S. AIC models will be shared. Continued research is needed to address the challenges of engaging older adults with lower socio-economic status, meeting older adults' diverse and dynamic needs, and conducting comparative studies to share lessons learned across the globe.

